# Preparation of PVA–CS/SA–Ca^2+^ Hydrogel with Core–Shell Structure

**DOI:** 10.3390/polym14010212

**Published:** 2022-01-05

**Authors:** Shuai Zhang, Yu Wan, Weijie Yuan, Yaoxiang Zhang, Ziyuan Zhou, Min Zhang, Luzhen Wang, Ran Wang

**Affiliations:** 1 Experimental Center of Forestry in North China, Chinese Academy of Forestry, Beijing 102300, China; zhang.s@caf.ac.cn (S.Z.); wanyu96115a@163.com (Y.W.); zyx631022@263.net (Y.Z.); zhouziyuan@caf.ac.cn (Z.Z.); zhanglaoshi00@163.com (M.Z.); wangran@caf.ac.cn (R.W.); 2 National Permanent Scientific Research Base for Warm Temperate Zone Forestry of Jiulong Mountain in Beijing, Beijing 102300, China; 3 School of Environmental Science and Engineering, Shaanxi University of Science and Technology, Xi’an 710021, China; 4 Qinghai Provincial Investigation, Design &Research Institute of Water Conservancy & Hydropower Co., Ltd., Xining 810000, China; wlzroger@163.com

**Keywords:** core–shell structure, hydrogel, double load, response surface

## Abstract

Hydrogels are highly hydrophilic polymers that have been used in a wide range of applications. In this study, we prepared PVA–CS/SA–Ca^2+^ core–shell hydrogels with bilayer space by cross-linking PVA and CS to form a core structure and chelating SA and Ca^2+^ to form a shell structure to achieve multiple substance loading and multifunctional expression. The morphology and structure of core–shell hydrogels were characterized by scanning electron microscopy (SEM) and Fourier transform infrared spectroscopy (FTIR). The factors affecting the swelling properties of the hydrogel were studied. The results show that the PVA–CS/SA–Ca^2+^ hydrogel has obvious core and shell structures. The SA concentration and SA/Ca^2+^ cross-linking time show a positive correlation with the thickness of the shell structure; the PVA/CS mass ratio affects the structural characteristics of the core structure; and a higher CS content indicates the more obvious three-dimensional network structure of the hydrogel. The optimal experimental conditions for the swelling degree of the core–shell hydrogel were an SA concentration of 5%; an SA/Ca^2+^ cross-linking time of 90 min; a PVA/CS mass ratio of 1:0.7; and a maximum swelling degree of 50 g/g.

## 1. Introduction

Hydrogels are a class of hydrophilic polymers with a three-dimensional network structure formed by cross-linking with the action of covalent and hydrogen bonds [1,2,3], which have a large specific surface area [4], high carrier strength [5], controllability [6] and a wide range of physicochemical adjustability [7,8]. Since the 1960s, when the Czech scholar Wichterle first produced poly (2-hydroxyethyl methacrylate) hydrogels [9], hydrogels have been widely used in medicine, engineering, agriculture, forestry, environmental protection and the information industry due to their good biocompatibility, sensitivity to environmental changes and superb molecular designability [10,11,12,13,14,15].

Hydrogel is a material similar to the tissue of living organisms, which has a wide range of medical applications because of its excellent biological properties. It can both encapsulate drugs for slow release into the body and load dressings for contact with tissue wounds [16]. High-strength composite hydrogels can also be used as scaffold materials [17]. The high hydrophilicity of hydrogel can hold a large amount of water, play the role of water retention and drought prevention and promote the growth of crops and forests [18]; hydrogel has a strong adsorption effect and can adsorb heavy metals and pollutants in the soil [19]; hydrogel can also remove a wide range of aqueous pollutants containing toxic dyes and organic pollutants [20,21]. With the development of artificial intelligence, hydrogel strain sensors can be produced, which have enabled the extensive development of flexible wearable devices [22]. Due to its own properties, hydrogel can take on important roles. In addition, it can respond to changes in the external environment, for example, when factors such as temperature and humidity, pH and light intensity change, the three-dimensional network structure of the hydrogel will also change in response, which is why hydrogels are also known as smart polymers [23]. In addition, the composite of hydrogel and different media will expand its application areas, for example, the introduction of hydrogel into conductive media can produce conductive hydrogel [24].

The properties of hydrogels are determined by the polymer network structure. Single-material hydrogels are isotropic in terms of microstructure and macroscopic properties, lacking an ordered structure, and have poor mechanical properties, which in turn limit their application in some fields. A double network structure is usually used to solve the mechanical property problem, and a biocompatible material is used to solve the problem of poor biocompatibility. Double network hydrogels usually contain two kinds of networks: one is rigid, which is easy to break and can increase the tensile stress of the hydrogel by dissipating energy; and the other is flexible, which can increase the tensile strain of the hydrogel [25,26]. The polyvinyl alcohol (PVA) hydrogel is a class of highly hydrophilic, non-toxic, degradable elastomeric materials with good film formation and chemical stability, but a single PVA hydrogel is prone to swelling and adhesion and poor stability. Chitosan (CS) has good biocompatibility and tunability, but the intermolecular hydrogen bonding force is strong, and when it is solely used to prepare hydrogels, the products suffer from disadvantages such as their brittleness and poor mechanical properties. The cross-linking of PVA material with CS can make up for the deficiencies of both materials [27]. Sodium alginate (SA) is a green biomass sodium salt with strong affinity to dyes and metal ions because of its abundant hydroxyl and carboxyl groups, but the mechanical strength of a single SA hydrogel is weak, while cross-linking with Ca^2+^ through chelation can significantly enhance the stability of the structure [28].

Previous studies of hydrogels have shown them to be versatile but only able to perform a specific function. In this study, we propose to use polyvinyl alcohol (PVA) and chitosan (CS) to build the core of the hydrogel, and sodium alginate (SA) and Ca^2+^ to construct the shell, forming a core–shell structure with a bilayer space that does not affect each part of the structure in order to achieve the purpose of loading two substances, so that one hydrogel can achieve two different functions.

## 2. Experimental Method

### 2.1. Raw Materials and Reagents

Polyvinyl alcohol (PVA) was purchased from Sinopharm Group Chemical Reagent Co., (Shanghai, China, AR); chitosan (CS) and sodium alginate (SA) were purchased from Chengdu Kelong Chemical Reagent Factory, (Chengdu, China, AR); calcium chloride was purchased from Tianjin Tianli Chemical Reagent Co., (Tianjin, China, AR); citric acid was purchased from Tianjin Comio Chemical Reagent Co., (Tianjin, China, AR); dipotassium hydrogen phosphate was purchased from Tianjin Chemical Reagent Factory, (Tianjin, China, AR); sodium dihydrogen phosphate was purchased from Tianjin Fuchen Chemical Reagent Factory, (Tianjin, China, AR); and D-(+)-gluconic acid delta-lactone was purchased from Shanghai Maclean Biochemical Co., (Shanghai, China, AR).

Centrifuge (80-1), Shanghai Pudong Physical Optical Instrument Factory (Shanghai, China); freeze dryer (LGJ-10), Beijing Songyuan Huaxing Technology Development Co. (Beijing, China); scanning electron microscope (FEI Q45), FEI Inc. (Hillsboro, OR, USA); X-ray photoelectron spectroscopy (Vario EL III), Kratos Analytical Ltd. (Manchester, UK); Fourier transform infrared spectroscopy (VECTOR-22), Bruker Co. (Karlsruhe, Germany).

### 2.2. Preparation of PVA–CS/SA–Ca^2+^ Core–Shell Hydrogels

**Figure 1:**
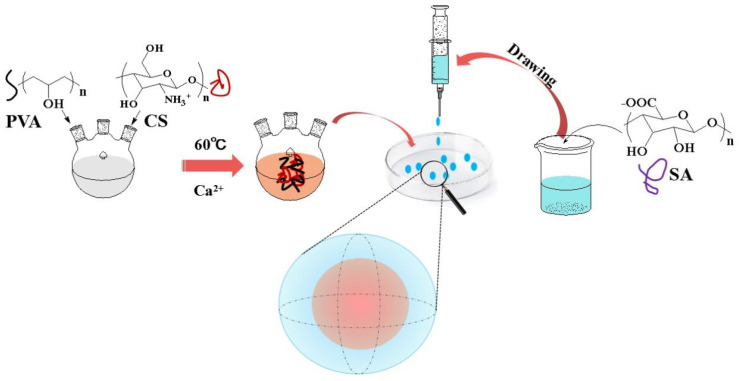
Preparation of PVA–CS/SA–Ca^2+^ core–shell hydrogel.

A PVA solution with a mass fraction of 10% was prepared by dissolving 1 g of PVA in 10 mL of distilled water and mechanically stirring at 120 rpm for 2 h in a water bath at 90 °C. Subsequently, 0.4 g of CS and citric acid were dispersed in 10 mL of distilled water at 20 °C and mechanically stirred at 120 rpm for 4 h to obtain a CS suspension with a mass fraction of 4%. Then, different volumes of CS suspension were added to the PVA solution and mechanically stirred at 180 rpm for 1 h at 60 °C. Moreover, 1 g of CaCl_2_ and 1 g of gluconolactone were added to the reaction system and stirred for 30 min before 1 mL of different concentrations of SA solution was pipetted into the above mixed solution and left for different times to form hydrogels. The obtained hydrogels were referred to as PVA–CS/SA–Ca^2+^ core–shell hydrogels. Finally, the dried core–shell hydrogels were obtained by cyclic freeze–thawing 3 times and then washed several times with distilled water and vacuum freeze-dried and stored in a drying tower for use, as shown in Figure 1.

The amounts of CS, SA and SA/Ca^2+^ cross-linking time used to prepare the hydrogels are listed in Table 1.

### 2.3. Structural Characterization and Performance Testing of PVA–CS/SA–Ca^2+^ Core–Shell Hydrogels

#### 2.3.1. Microscopic Morphology of PVA–CS/SA–Ca^2+^ Core–Shell Hydrogels (SEM)

The core–shell structure of the freeze–thawed core–shell hydrogel was separated and freeze-dried under vacuum, after which it was cut into thin slices with a thickness of 0.5 mm and sprayed with gold at a test voltage of 25 kV to a thickness of 10 nm. The microscopic morphology of the dried core–shell samples was observed using an FEI Q45 scanning electron microscope.

#### 2.3.2. Structure of PVA–CS/SA–Ca^2+^ Core–Shell Hydrogels (FTIR)

The core–shell structure of the core–shell hydrogel after vacuum drying for 24 h was characterized by using a VECTOR-22 Fourier transform infrared spectrometer with a set wavelength range of 500~4000 cm^−1^ and 32 scans.

#### 2.3.3. Swelling Properties of PVA–CS/SA–Ca^2+^ Core–Shell Hydrogels

The effects of SA concentration, SA and Ca^2+^ cross-linking time and PVA/CS mass ratio on the swelling of PVA–CS/SA–Ca^2+^ core–shell hydrogels were investigated to determine the range of each parameter and to provide a basis for optimizing the process parameters for the swelling of core–shell hydrogels. The freeze-dried core–shell hydrogels were weighed (W_0_), added to 7 mL of disodium hydrogen phosphate at a concentration of 1/15 mol/L and 3 mL of potassium dihydrogen phosphate buffer at a concentration of 1/15 mol/L, and left to absorb water at 25 °C, removed at intervals of 20 min, dried on moist filter paper and precisely weighed (W_t_)—all of which was repeated several times until the swelling equilibrium was reached, and the experiments were conducted in parallel nine times. The swelling degree (SR) of the core–shell hydrogel was calculated according to the following equation [29]:

$$\mathrm{SR}=\frac{W_{t}-W_{0}}{W_{0}}$$

where W_0_ and W_t_ are the initial dry weight (g) and the mass (g) of the sample at t min of water absorption, respectively; SR (g/g) is the swelling degree of hydrogel.

### 2.4. Statistical Analysis

The statistical analyses were performed by the SPSS computer program (SPSS Statistic 20.0) software using one-way analysis of variance (ANOVA). Values were presented as means ± standard deviations (SD) of triplicate determinations. Statistical significance was set at *p* < 0.05.

## 3. Results and Analysis

### 3.1. Microscopic Morphology of PVA–CS/SA–Ca^2+^ Core–Shell Hydrogels

**Figure 2:**
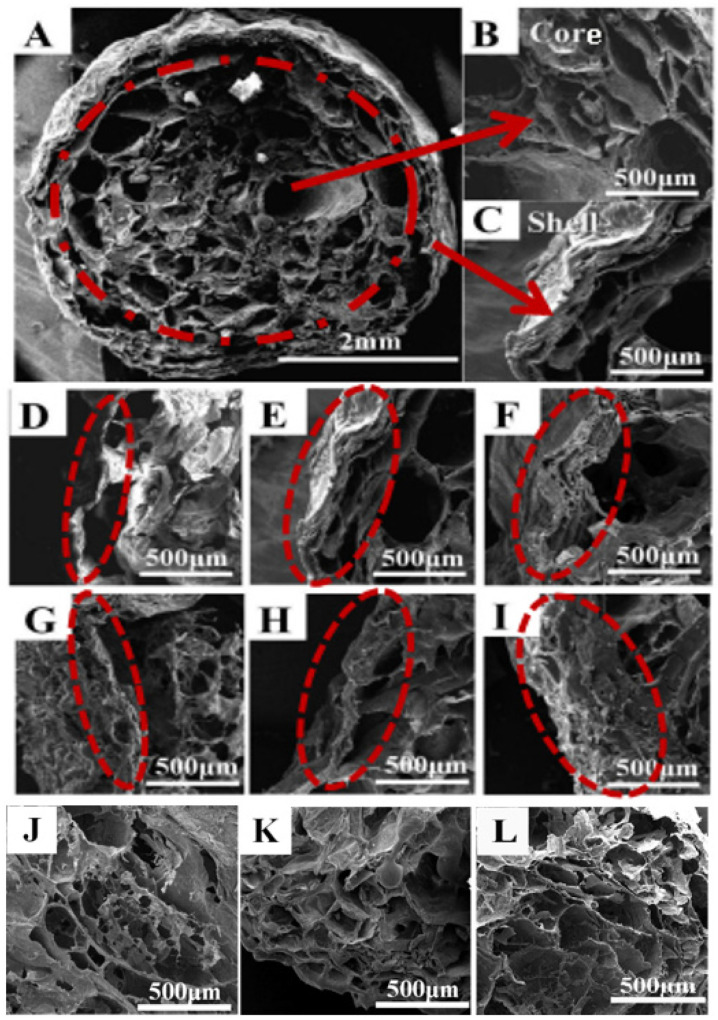
SEM photograph of PVA–CS/SA–Ca^2+^ core–shell hydrogel ((**A**–**C**) are core–shell hydrogels with 4% SA, PVA/CS = 1:0.8, and SA/Ca^2+^ cross-linking time of 30 min; (**D**–**F**) are core–shell hydrogels with 4% SA, PVA/CS = 1:0.8, and SA/Ca^2+^ cross-linking time of 30 min, 60 min and 90 min; (**G**–**I**) are core–shell hydrogels with PVA/CS = 1:0.8, SA/Ca^2+^ cross-linking time of 30 min, and SA concentrations of 2%, 4% and 6%, respectively; (**J**–**L**) are core–shell hydrogels with 4% SA, SA/Ca^2+^ cross-linking time of 30 min, and PVA/CS of 1:0.6, 1:0.8 and 1:1).

The SEM photographs of PVA–CS/SA–Ca^2+^ core–shell hydrogel is shown in Figure 2. The surface of the hydrogel is rough with many folds, which can provide a larger surface area. Here, A is a cross-sectional view of the core–shell structure, and B and C are the core and shell structures, respectively. The core structure of the hydrogel is a relatively loose and three-dimensional network structure with more pores, and the shell structure is relatively compact with fewer pores, which is mainly because the network structure formed by the cross-linking of PVA and CS molecular chains is looser, while the chelating force of SA and Ca^2+^ is strong and the chelating rings formed are tightly connected, so the shell structure formed is denser. As shown in Figure 2D–F, with the increase in cross-linking time, the shell wall of the core–shell hydrogel becomes thicker and the pore structure increases, which indicates that with the increase in cross-linking time, the reaction between SA and Ca^2+^ will be more complete and the three-dimensional structure will be more stereoscopic and stable. In Figure 2G–I, the hydrogel shell walls become thicker and denser with the increasing SA concentration, which indicates that the increase in SA concentration makes more SA coordination atoms form chelate rings with Ca^2+^ and occupy the pores of the 3D network structure. As shown in Figure 2J–L, the three-dimensional network structure of the hydrogel core structure becomes more and more significant as the PVA/CS mass ratio decreases. These results indicate that the SA/Ca^2+^ cross-linking time and SA concentration have a strong influence on the shell structure of hydrogels, and a longer cross-linking time means a larger SA concentration, a thicker hydrogel shell wall and a more stable structure; the PVA/CS mass ratio can regulate the spatial conformation of the hydrogel nuclear structure. The structure of the hydrogel determines its properties, and the properties determine its usage, so a different PVA/CS mass ratio, SA/Ca^2+^ cross-linking time and SA concentration can be set according to the different usage of core–shell hydrogel to achieve the purpose of controlling the core–shell structure of hydrogel.

### 3.2. Structure of Core–Shell Hydrogel

**Figure 3:**
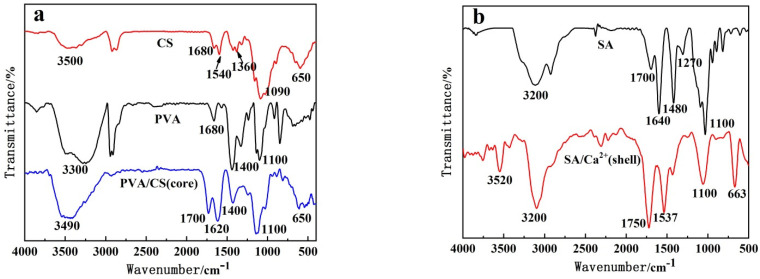
FTIR spectra of PVA–CS/SA–Ca^2+^ core–shell hydrogel and its raw materials: (**a**) shows the FTIR spectra of PVA/CS nuclear structure and its raw materials PVA and CS; (**b**) shows the FTIR spectra of SA/Ca^2+^ shell structure and its raw material SA.

Figure 3 shows the FTIR spectra of the PVA–CS/SA–Ca^2+^ core–shell hydrogel (4% SA, SA/Ca^2+^ cross-linking time 30 min, PVA/CS = 1:0.6) and its raw materials. In Figure 3a, 3500 cm^−1^ shows the superposition peaks of the amino (-NH) and hydroxyl (-OH) groups of CS, 1360 cm^−1^, 1090 cm^−1^ and 650 cm^−1^ which show the out-of-plane bending vibration absorption peaks of carbon and nitrogen bonds (C–N), ether groups (C–O–C) and N–H of CS, respectively, and 1680 cm^−1^ and 1590 cm^−1^, which show the characteristic absorption peaks of the undeacylated carbonyl group of CS (C=O) characteristic absorption peaks. Additionally, 3300 cm^−1^, 1680 cm^−1^, 1400 cm^−1^ and 1100 cm^−1^ are the absorption peaks of -OH, unpolymerized carbon–carbon double bond (–C=C–), alkyl and carbon–oxygen single bond (C–O) of PVA, respectively. Compared with the spectra of CS and PVA, no new characteristic peaks appear in PVA/CS, except that the peaks of -NH and –OH of CS at 3500 cm^−1^ are shifted to 3490 cm^−1^, indicating that there is no chemical reaction between PVA and CS, but a hydrogen bond cross-linking between –NH or –OH of CS and –OH of PVA. In addition, the C=O absorption peaks of CS at 1680 cm^−1^ and 1540 cm^−1^ were shifted to 1700 cm^−1^ and 1620 cm^−1^, respectively, and the intensity of the peaks also significantly increased, which might be due to the reaction between the core and shell structures of the hydrogel and the introduction of the carboxyl group of SA.

In Figure 3b, the absorption peaks of SA are O–H, C–O, and C–O–C at 3200 cm^−1^, 1270 cm^−1^ and 1100 cm^−1^, respectively, and the absorption peaks of C=O and -OH are at 1700 cm^−1^, 1640 cm^−1^ and 1480 cm^−1^, respectively. The -OH peak of SA in the SA/Ca^2+^ shell structure has a narrowed peak shape and weakened intensity, while the C=O peaks changed and shifted, mainly due to the chelation of SA –OH and –COOH with Ca^2+^ to form the shell structure. The absorption peaks at 1520 cm^−1^ and 663 cm^−1^ were the same as those at 1360 cm^−1^ (C–N) and 650 cm^−1^ (N–H) in Figure 3a, respectively, indicating that the core and shell structures of the hydrogel were cross-linked through the hydrogen bonding between the CS amino group and the SA carboxyl group, which confirmed the above suspicion.

### 3.3. Swelling Properties of PVA–CS/SA–Ca^2+^ Core–Shell Hydrogels

#### 3.3.1. Single Factor Screening for Swelling Performance

(1) The effect of SA concentration.

**Figure 4:**
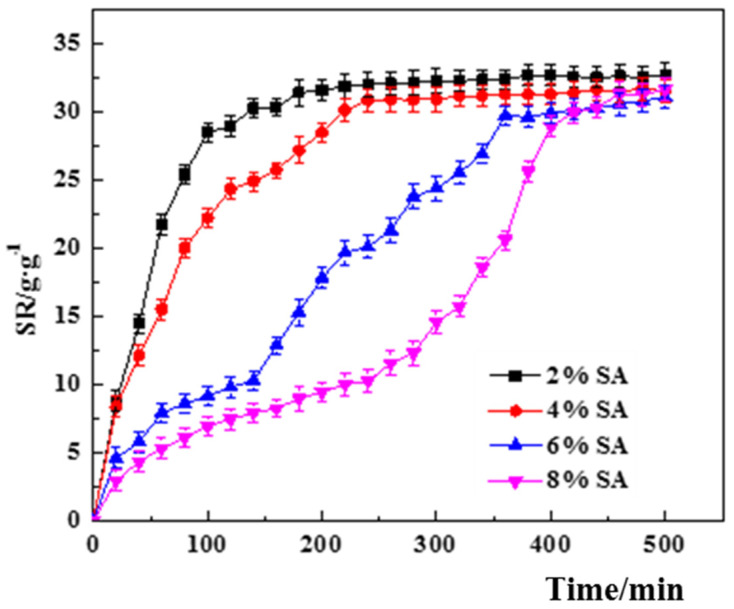
Effect of SA concentration on the swelling degree of PVA–CS/SA–Ca^2+^ core–shell hydrogel.

Figure 4 shows the variation graphs of the swelling degree of PVA–CS/SA–Ca^2+^ core–shell hydrogels at different SA concentrations. The maximum swelling of the core–shell hydrogels with different SA concentrations differed slightly, but the equilibrium swelling time was significantly different, showing a significant increase with the increase in SA concentration, indicating that the increase in SA concentration reduces the water absorption efficiency of hydrogels. Combined with the results of SEM observation (Figure 2G–I), the increase in SA concentration increases the cross-linkage with Ca^2+^, which leads to a thicker shell wall and denser structure, and decreases the pore area, rendering it difficult for water to enter the hydrogel, and the equilibrium swelling time increases.

(2) Effect of SA/Ca^2+^ cross-linking time.

**Figure 5:**
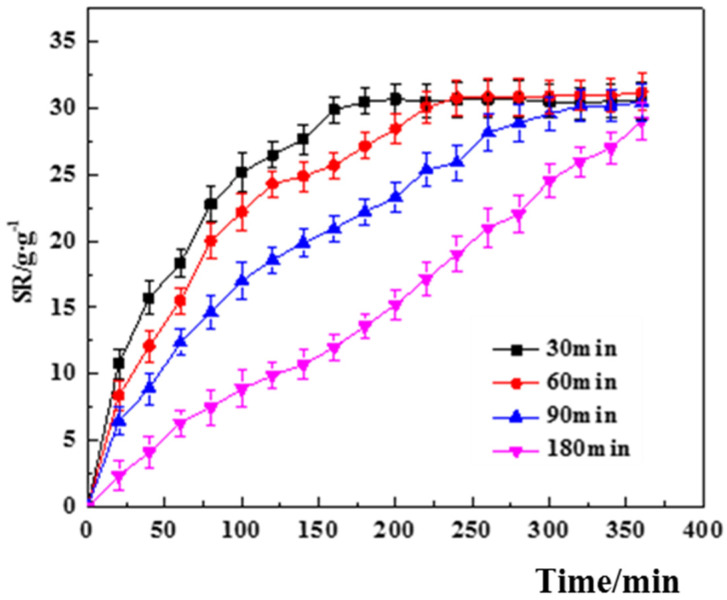
Effect of SA/Ca^2+^ cross-linking time on the swelling degree of PVA–CS/SA–Ca^2+^ core–shell hydrogel.

Figure 5 shows the variation curves of the swelling degree of the core–shell hydrogel under different cross-linking times of SA/Ca^2+^. The final equilibrium swelling of the core–shell hydrogel was basically the same at approximately 30 ± 1.4 g/g for different cross-linking times in the figure, but the equilibrium swelling time became longer and longer with the increase in cross-linking time, which was similar to the effect of SA concentration on the swelling of the core–shell hydrogel. This is because the cross-linking time of SA/Ca^2+^ affects the wall thickness of the core–shell hydrogel, and a longer cross-linking time means a thicker hydrogel shell wall, leading to an increasingly longer swelling time.

(3) Effect of PVA/CS ratio.

**Figure 6:**
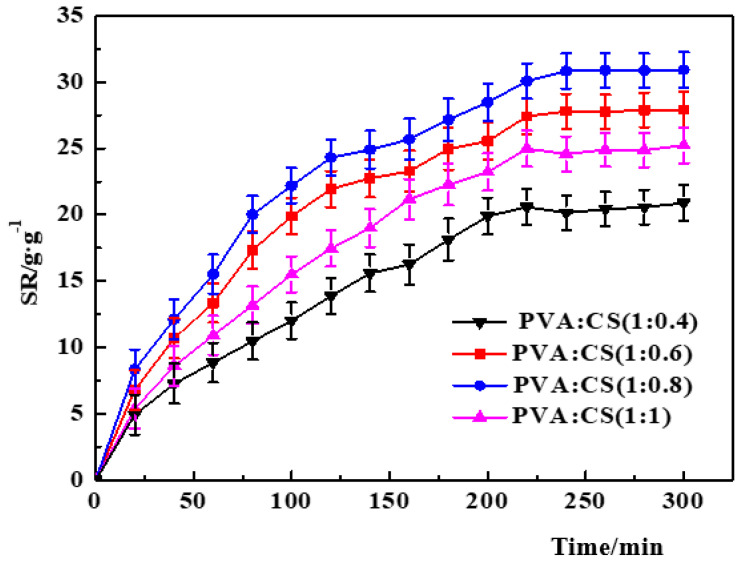
Effect of the PVA/CS ratio on the swelling degree of PVA–CS/SA–Ca^2+^ core–shell hydrogel.

Figure 6 shows the variation curves of the swelling degree of PVA–CS/SA–Ca^2+^ core–shell hydrogels with different PVA/CS mass ratios. With the increase in CS content, the swelling degree of the core–shell hydrogel shows a trend of increasing and then decreasing. When the PVA/CS mass ratio is 1:0.8, the swelling of the hydrogel is the largest, reaching 32 ± 1.4 g/g. This is due to the strong hydrogen bonding in CS, which can cross-link with PVA to form a more stable three-dimensional network skeleton when the content increases, which can also be reflected by the SEM results (Figure 2J–L). At the same time, CS contains many hydrophilic functional hydroxyl and carboxyl groups, which improve the water absorption of hydrogel. However, when the CS content is excessive, the three-dimensional network structure formed by cross-linking will be more compact, which reduces the surface pore space and affects the entry of water molecules.

#### 3.3.2. Response Surface Analysis

From Section 3.3.1, it can be concluded that the SA concentration, SA/Ca^2+^ cross-linking time and the PVA/CS mass ratio all affect the maximum swelling of PVA–CS/SA–Ca^2+^ core–shell hydrogels as well as the equilibrium swelling time; so in order to further investigate the magnitude of the effect of the three factors on the swelling of the core–shell hydrogels and the optimal conditions for the swelling of core–shell hydrogels, a response surface analysis was performed. The response surface analysis was performed.

(1) Response modeling.

Based on the experiments with the PVA–CS/SA–Ca^2+^ core–shell hydrogel swelling degree as the one-way variable method combined with the Box–Behnken central combination test design principle, the SA concentration (A), SA/Ca^2+^ cross-linking time (B) and PVA/CS mass ratio (C) were selected as independent variables and the swelling degree of core–shell hydrogel as the response value, and Table 2 shows the experimental factor levels.

(2) Model significance and response surface analysis.

Using Design Expert 10 software to optimize the experimental design and processing of the swelling degree of the core–shell hydrogel, according to the optimized results, the influence of the constant term, linear term, interaction term and square term on the swelling degree of the hydrogel can be obtained, and the analysis of the experimental results is quadratically fitted to obtain the quadratic multinomial regression equation between the swelling degree of the core–shell hydrogel (response value SR) and each influencing factor:

SR = −301.834 − 54.867 × A − 1.62499 × B+ 970.199 × C + 0.313121 × A × B + 15.5803 × A × B − 0.130687 × A × C + 0.237412 × A^2^ − 0.00146488 × B^2^ − 425.35 × C^2^

Table 3 shows the ANOVA results of the quadratic regression model, from which it can be seen that the effect of the model on the swelling degree of the core–shell hydrogel is highly significant (*p* < 0.01), and the experimental and predicted values are highly correlated (R^2^ = 0.9810, R^2^_adj_ = 0.9566), indicating that the model fits well experimentally and can be used to predict the optimal conditions for the swelling degree of the core–shell hydrogel. The degree of influence of each factor on the swelling degree of the core–shell hydrogel was inferred from the magnitude of F value as: SA/Ca^2+^ cross-linking time (B) > PVA/CS mass ratio (C) > SA concentration (A). The primary terms A, B, C, interaction term AC and secondary term C2 of this model were significant (*p* < 0.05), and the interaction term BC reached a highly significant level (*p* < 0.01), indicating that the swelling of the core–shell hydrogels increased with the increase in SA concentration and SA/Ca^2+^ cross-linking time.

**Figure 7:**
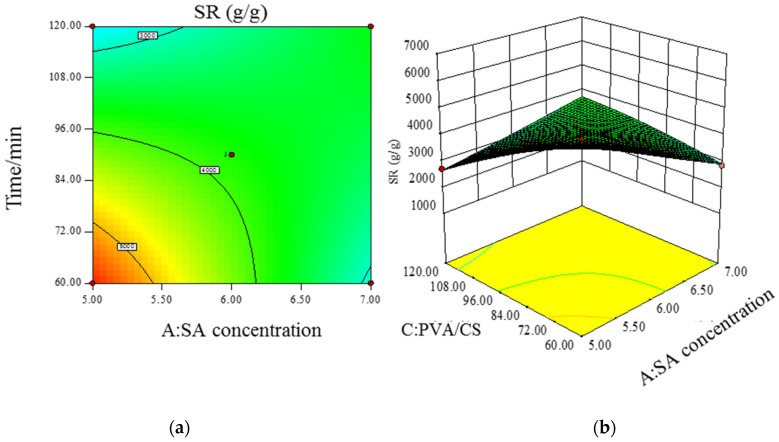
Contour (**a**) and 3D plot (**b**) of SA concentration versus SA/Ca^2+^ cross-linking time interaction versus response value SR.

**Figure 8:**
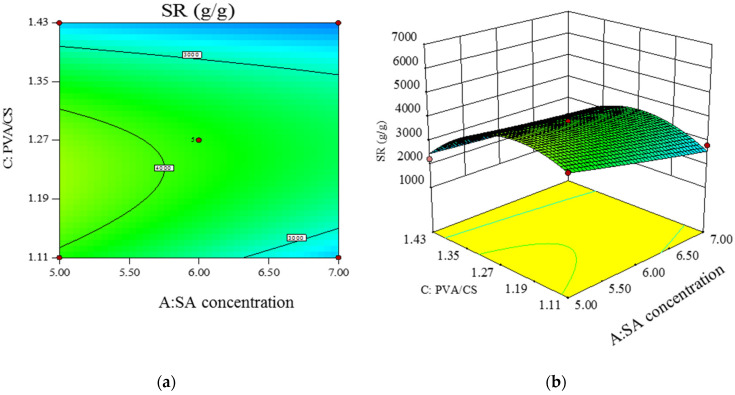
Contour (**a**) and 3D plot (**b**) of SA concentration versus PVA/CS addition ratio interaction versus response value SR.

**Figure 9:**
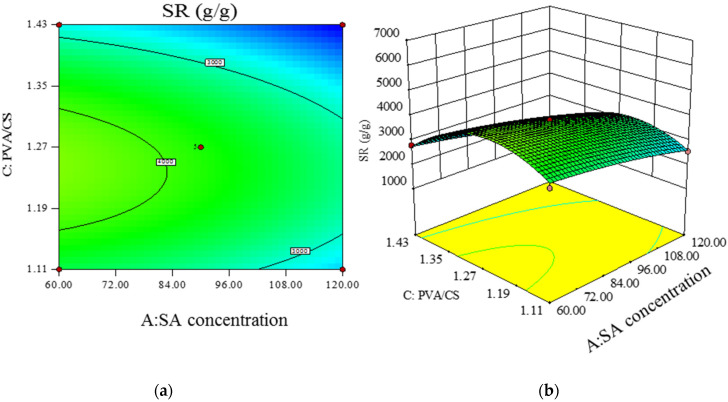
Contour (**a**) and 3D plot (**b**) of SA/Ca^2+^ cross-linking time versus PVA/CS addition ratio interaction versus response value SR.

Figure 7 shows the contour (a) and three-dimensional plot (b) of the interaction between SA concentration and SA/Ca^2+^ cross-linking time on the swelling degree of response values. From Figure 7a, it can be seen that the response surface is relatively flat and the contours are approximately circular, and the changes of hydrogel swelling are not obvious with the increase in cross-linking time when the SA concentration is at low and high values. This indicates that the interaction between SA concentration and SA/Ca^2+^ cross-linking time is not significant. Figure 8 shows the contour (a) and 3D plot (b) of the interaction between SA concentration and PVA/CS mass ratio on the swelling degree of the response value. The contour in Figure 8a is elliptical, and the swelling degree increases and then decreases with the increase in PVA/CS mass ratio when the SA concentration is at low and high values. This indicates that the interaction between SA concentration and PVA/CS mass ratio is more significant. Figure 9 shows the contour (a) and 3D plot (b) of the interaction between the SA/Ca^2+^ cross-linking time and the PVA/CS mass ratio on the swelling degree of the response value, the contour in Figure 9a is elliptical, and the swelling degree increases and then decreases with the increase in PVA/CS mass ratio when the cross-linking time is at low and high values. This indicates that the interaction of the cross-linking time and PVA/CS mass ratio has a significant effect on the swelling degree.

(3) Prediction of optimal experimental conditions for swelling degree.

We can see from the predicted results that the optimal experimental conditions for the core–shell hydrogel are an SA concentration of 5%, SA/Ca^2+^ cross-linking time of 90 min, and PVA/CS mass ratio of 1:0.7, when the maximum swelling degree can be obtained, which is approximately 50 g/g. To verify this conclusion, we conducted experimental measurements of the swelling degree under these experimental conditions, and the maximum swelling degree results obtained were 49 ± 1.2 g/g, which is in general agreement with the predicted results.

## 4. Discussions

### 4.1. Swelling Properties of Hydrogels

PVA and SA are commonly used cross-linking materials for hydrogels, and a large number of different types of hydrogels have been explored by previous studies for the preparation and swelling performance testing. The maximum swelling of CS/PVA hydrogels prepared by Luo et al. was approximately 20 g/g [30]; the maximum swelling of sodium alginate/polyvinyl alcohol (SA/PVA) hydrogels prepared by Ji et al. using the freeze–thaw method was approximately 10 g/g, and it could be increased to approximately 20 g/g when calcium alginate/polyvinyl alcohol (CA/PVA) was prepared using the Ca^2+^ release method and the freeze–thaw cycling method [31]. Polymalic acid is the only kind of high molecular polymer that contains a fat bond and can be quickly hydrolyzed, and the hydrolysis product is malic acid monomer, which can be degraded in the tricarboxylic acid cycle. The hydrogel prepared with PVA reaches a swelling equilibrium after 24 h water absorption, and the maximum swelling degree is approximately 10.5 g/g [32]. In addition, environmental factors also affect the swelling properties of hydrogels. The maximum swelling rate of sodium alginate/graphene oxide hydrogels prepared by Zhuang et al. was approximately 15 g/g under neutral conditions, but the maximum swelling rate can grow to 35 g/g when adjusting the pH to 13 [33]; Hülya found in his study that temperature affects the swelling of hydrogels, and the swelling rate is at its highest at 40 °C [34].

The swelling property is one of the most important parameters for evaluating the structure of hydrogels, and a high swelling degree reduces the stability and mechanical strength of hydrogels. In general, hydrogels that can be used repeatedly tend to limit the maximum swelling degree, except for disposable products such as diapers and hemostats that can be used with high swelling degree hydrogels. The mechanical strength of hydrogels depends on the cross-linking density, so the appropriate cross-linking conditions can be selected according to specific needs.

### 4.2. Dual Loading Function of PVA–CS/SA–Ca^2+^ Core–Shell Hydrogels

The main objective of this study was to construct the core–shell structure of hydrogel for the purpose of loading two substances for multifunctional expression. After preparing the core–shell hydrogel, in order to verify the dual loading function of the hydrogel, we added degradation bacteria in the core structure and photocatalyst in the shell structure, taking methylene blue (MB) as the research object, and analyzed the degradation of MB. The results showed that MB was first degraded by the degradation bacteria after entering the hydrogel, and the color of the solution becomes lighter. When the light is irradiated through the solution to the hydrogel, MB was further degraded by the photocatalyst. The core–shell hydrogel showed a gradual degradation function, which indicates that the core–shell hydrogel can achieve multi-functional expression.

## 5. Conclusions

(1) The PVA–CS/SA–Ca^2+^ hydrogels have obvious nucleation and shell structures as observed by SEM. The SA concentration and SA/Ca^2+^ cross-linking time are positively correlated with the thickness of the shell structure; the PVA/CS mass ratio affects the structural characteristics of the nucleation structure, and a higher CS content means a more obvious three-dimensional network of the hydrogels’ structure.
(2) The optimal experimental conditions for the swelling of the core–shell hydrogels were obtained by single-factor screening and surface response analysis, which were an SA concentration of 5%, SA/Ca^2+^ cross-linking time of 90 min, PVA/CS mass ratio of 1:0.7 and maximum swelling of 50 g/g.

## Figures and Tables

**Table 1:** Reaction conditions of PVA–CS/SA–Ca^2+^ core–shell hydrogels.

Sample	PVA/mL	CS/mL	SA/%	SA/Ca^2+^ Cross-Linking Time/min
1	10	20	4	30
2	10	20	4	60
3	10	20	4	90
4	10	20	2	30
5	10	20	6	30
6	10	15	4	30
7	10	25	4	30

**Table 2:** Factors and levels for Box–Behnken design.

Level	SA Concentration	SA/Ca^2+^ Cross-Linking Time	PVA/CS
1	5	60	1:0.7
2	6	90	1:0.8
3	7	120	1:0.9

**Table 3:** Analysis of variance for quadric regression model.

Variation Source	Sum of Squares	Freedom	Mean Square	F Value	*p* Value	Significance
Model	1414.60	9	157.18	40.20	<0.0001	**
A	131.263	1	131.23	33.56	0.0007	**
B	222.81	1	222.81	56.98	0.0001	**
C	166.01	1	166.01	42.45	0.0003	**
AB	352.96	1	352.96	0.40	0.5460	
AC	24.86	1	24.86	6.36	0.0397	*
BC	1.57	1	1.57	90.26	<0.0001	**
A^2^	0.24	1	0.24	0.061	0.8125	
B^2^	7.32	1	7.32	1.87	0.2136	
C^2^	499.24	1	499.24	127.67	<0.0001	**
Residual	27.37	7	3.91			
Spurious term	27.37	3	9.12			
Pure error	0.00	4	0.00			
Total value	1441.98	16				
Model determination coefficient	R^2^ = 0.9810					
Model adjustment determination Coefficient	R^2^_adj_ = 0.9566					

Note: ** indicates highly significant (*p* < 0.01); * indicates significant (*p* < 0.05).
